# Commentary: “You're Only as Strong as Your Weakest Link”: A Current Opinion About the Concepts and Characteristics of Functional Training

**DOI:** 10.3389/fphys.2021.744144

**Published:** 2021-11-04

**Authors:** Bernardo N. Ide, Moacir Marocolo, Clarcson P. C. Santos, Bruno V. C. Silva, Amanda Piaia Silvatti, Mário Antonio Moura Simim, Dustin J. Oranchuk, Guilherme Goulart de Agostini, Gustavo R. Mota

**Affiliations:** ^1^Exercise Science, Health and Human Performance Research Group, Department of Sport Sciences, Institute of Health Sciences, Federal University of Triângulo Mineiro, Uberaba, Brazil; ^2^Physiology and Human Performance Research Group, Department of Physiology, Federal University of Juiz de Fora, Juiz de Fora, Brazil; ^3^Research Group on Metabolic Diseases, Physical Exercise and Health Technologies, Bahiana School of Medicine and Public Health, Salvador, Brazil; ^4^Department of Physical Education, University Center of Belo Horizonte, Belo Horizonte, Brazil; ^5^Department of Physical Education, Federal University of Viçosa, Viçosa, Brazil; ^6^Physical Education and Adapted Sports Research Group, Institute of Physical Education and Sports, Federal University of Ceará, Fortaleza, Brazil; ^7^Sports Performance Research Institute New Zealand, Auckland University of Technology, Auckland, New Zealand; ^8^Faculty of Physical Education, Federal University of Uberlândia, Uberlândia, Brazil

**Keywords:** exercise, resistance exercise, strength training, misconception, exercise physiologist

## Introduction

Exercise adaptations are highly dependent on the specific training stimulus (Nader, 2006; Egan and Zierath, 2013; Hughes et al., 2018). Therefore, an apt description of physical training programs is essential for adequate planning of neuromuscular, cardiovascular, metabolic, and functional exercise performance and recovery enhancements. Although muscle strength, power, flexibility, and endurance training are well-defined concepts within exercise prescription and muscle performance, functional training (FT) does not have a universal definition.

Examining the manuscript of La Scala Teixeira et al. (2017), reviled inconsistent concepts regarding the definition of FT. Specifically, they did not differentiate FT from strength, power, flexibility, or endurance training programs. In the PubMed database, their manuscript (La Scala Teixeira et al., 2017) was cited by six articles (Crawford et al., 2018; Da Silva-Grigoletto et al., 2019, 2020; Drum et al., 2019; La Scala Teixeira et al., 2019; Muyor et al., 2020), three of which were self-citations. Despite not yet (as of May 2021) being extensively cited in the literature, their manuscript (La Scala Teixeira et al., 2017) has a total of 9,535 views (https://www.frontiersin.org/articles/10.3389/fphys.2017.00643/full) and 1,282 downloads (http://loop-impact.frontiersin.org/impact/article/285700#totalviews/downloads), and may create a relevant social and health impact.

Considering that functional fitness training was regarded as one of the Top 20 Worldwide Fitness Trends for 2021 (Thompson, 2021), the dissemination of inconsistent concepts regarding the definition of FT may create substantial confusion among students, coaches, athletes, and sports scientists. In this context, this commentary builds upon the concepts and characteristics of FT presented by La Scala Teixeira et al. (2017). Therefore, we aimed to present a critical commentary to enrich the debate of such a topic and alleviate the potential confusion.

## The State of Definition

FT definition provided by the authors (La Scala Teixeira et al., 2017) was: “*The concept of FT is related to development of different physical capacities in an integrated and balanced manner in order to provide autonomy, efficiency and safety during activities related to daily living, work and/or sports. For this purpose, FT uses strength exercises generally characterized by integrated, multi-joint/multi-segment, asymmetrical, multi-planes, acyclic, intermittent, speedy, and unstable movements that emphasize core stability*”.

This statement presents some inconsistencies, and problems arise in the following domains:

1. What does “*in an integrated and balanced manner*” mean? The interference phenomenon with concurrent training presents attenuated muscle strength and mass gains compared to strength and power alone (Fyfe et al., 2014). Thus, how does FT provide different physical capacities in an “integrated and balanced manner”? Additionally, the authors (La Scala Teixeira et al., 2017) did not provide any scientific data to support “an integrated and balanced” development of different physical capacities.
2. If FT uses strength exercises, it could be easily defined as strength training. If strength exercises are combined with endurance exercises, a rational and more straightforward terminology could be “combined,” “concurrent training,” or mention the specific type of exercises performed (e.g., strength, plus endurance). Therefore, there is no need to “create” new terminology (i.e., FT) containing the aforementioned inconsistencies.
3. What is an “*integrated, asymmetrical, multi-planes, acyclic, intermittent, speedy, and unstable movement*”, strength exercise? To our knowledge, no definition nor example for this type of exercise is provided in their text or the broader literature.

In addition, the statement that FT provides “*autonomy, efficiency and safety during activities related to daily living, work and/or sports*” (La Scala Teixeira et al., 2017) generates another concern. All these benefits are already well-consolidated to the practice of traditional training programs (Cormie et al., 2011a,b; Buchheit and Laursen, 2013; Egan and Zierath, 2013; Baar, 2014; Hughes et al., 2018). Thus, it is not an exclusive or differentiating characteristic of FT programs *per se*.

Also, on the topic of “characteristic of FT,” the authors stated that “*in traditional combined training, two or more physical capacities are trained in the same session (e.g., strength and endurance) but at different times or with different exercises, while in FT these capacities are trained simultaneously, preferentially in the same exercise*” (La Scala Teixeira et al., 2017). This statement also presents some inconsistencies, and problems arise in the following domains:

1. Combined, or concurrent training, is defined as simultaneously incorporating both resistance and endurance exercise within a periodized training regime (Fyfe et al., 2014), and not just in the same session, as mentioned by the authors (La Scala Teixeira et al., 2017).
2. The word “*simultaneously”* was employed to describe an exclusive FT characteristic of developing strength and endurance in the same exercise. Although, the expressions “*at the same time”, “at one and the same time”, “at the same instant”, “at the same moment”, “at once”, “concurrently”, and “concomitantly*” are synonymous with “*simultaneously”* (https://www.lexico.com/synonyms/simultaneously). Since the authors employed the expression “*in the same session”* to describe a characteristic of traditional combined training, they failed in differentiating from FT. Besides, the simultaneous development in strength and endurance in the same exercise is already reported to the practice of traditional resistance training performed with high-volumes (e.g., two sets of 20–28 repetition maximum with 1 min rest interval) (Campos et al., 2002). Therefore, is not an exclusive characteristic of FT.

## Cited References Do Not Support Their Statements

In the “functional training: conceptual basis” topic (La Scala Teixeira et al., 2017), the work of Okada et al. (2011) is cited to support three statements. Although analyzing Okada's manuscript (Okada et al., 2011), we realize that it supports none of the statements. The cited study (Okada et al., 2011) aims to determine the relationships between core stability, functional movements, and performance and identify assessment tests that best predictor or represent motor performance. Twenty-eight healthy individuals performed tests in 3 categories: core stability, motor performance, and the functional movement screen (FMS). No significant correlations were observed between core stability and FMS scores. The weak- to moderate correlations observed (see Table 2 of their article) suggested that core stability and FMS are not strong predictors of motor performance in the evaluated tasks (Okada et al., 2011). Thus, besides not supporting the statements, the cited reference (Okada et al., 2011) concluded that functional movements and core stability training should not be the primary emphasis for increases in motor performance; the opposite of La Scala Teixeira's claim!

In the last paragraph of the “characteristics of functional training” topic (La Scala Teixeira et al., 2017), the works of Tomljanović et al. (2011) and Heinrich et al. (2012) are cited to support the following statement: “the focus on developing core stability is a marked feature of FT programs.” Tomljanović et al.'s study (Tomljanović et al., 2011) aimed to determine the effects of 5 weeks of FT and traditional strength training on anthropometric measures (body mass, fat percentage, lean body mass, and total body water), and neuromuscular performance tests (5-10-5 meter shuttle run and the hexagon test), jumping ability (air time, peak power, jump height, ground contact time), throwing ability tests (standing overarm medicine ball throw and lying medicine ball throw), and sprint variables (10 and 20 m dash and 10–20 m split time results) in young trained male subjects. Non-significant changes were observed for anthropometric measures. Significant improvements were observed in the medicine ball throw, shuttle run, and the hexagon test values for FT group. In contrast, traditional strength training significantly increases ground contact time, peak power, shuttle run, and the hexagon test performance, but decreases medicine ball throw performance. The authors concluded that FT and traditional strength training influenced neuromuscular performance differently.

Heinrich et al. (2012) aimed to compare the Mission Essential Fitness training to a standard Army Physical Fitness Test (APFT) program on fitness, physiological, and body composition changes. The Mission Essential Fitness program included “functional movements” focused on strength, power, speed, and agility. Pre-and post-measurements included the APFT, physiological indicators, body composition, and additional fitness indicators. The main results were that Mission Essential Fitness participants significantly increased their push-ups, bench press, and flexibility performance. Although, when compared to APFT participants, they decreased their 2-mile run and step test heart rate. The authors stated that the Mission Essential Fitness training is focused on increasing core stability. However, this aspect (i.e., core stability) was not evaluated in the study. Thus, the cited article (Heinrich et al., 2012) does not support the statement that “the focus on developing core stability is a marked feature of FT programs” (La Scala Teixeira et al., 2017).

In addition, it appears that the term “core stability” has no clear definition (Wirth et al., 2017). Most exercise specifications have not been tested for effectiveness, nor compared with the intensity specifications normally used for strength training (Wirth et al., 2017). Increased stabilization appears to be the result of increasing muscle strength (Wirth et al., 2017). Thus, if “core stability” is the desire for adaptation, classical strength-training exercises should be the basic stimuli (Wirth et al., 2017).

## Rational Statement: Functional Training Is not Different From Traditional Strength, Power, Flexibility, and Endurance Training

The definitions of FT presented by La Scala Teixeira et al. (2017) make the differentiation from traditional strength, power, flexibility, and endurance training programs difficult. The FT description provided in Figure 1A of their article refers to the increase in functional aspects of the neuromuscular system that could be defined as strength, power, endurance, and flexibility. Nevertheless, Figure 1B is misleading as it presents only strength exercises as a characteristic of FT.

Based on Figure 1 (La Scala Teixeira et al., 2017), it is crucial to clarify the basic mechanical concepts used in the description of muscular activity function: force, work, and power (Knuttgen, 1978), and in the classification of training stimuli/programs. They can be calculated according to the Newtonian mechanical concepts of displacement, velocity, and acceleration. Strength is the force developed by the muscles performing a particular joint movement (e.g., elbow flexion, knee extension) (Knuttgen and Komi, 2003). Work is expressed when the point of application of the force moves through displacement. Power is the rate of performing work; the derivative of work concerning time; the product of force and velocity (Knuttgen, 1978). In addition, muscles can maintain a specific isometric force or power level, involving combinations of concentric and eccentric muscular actions, a functional property known as endurance (Winter and Fowler, 2009). Flexibility is the intrinsic property of body tissues that determines the range of motion achievable without injury (Knudson et al., 2000).

Overall, the relevant authors (La Scala Teixeira et al., 2017) state that FT programs aim at developing functional aspects of the neuromuscular system to increase efficiency and safety during activities related to daily living, work, and sports. Although, all these benefits are already well-consolidated to the practice of traditional training programs (Cormie et al., 2011a,b; Buchheit and Laursen, 2013; Egan and Zierath, 2013; Baar, 2014; Hughes et al., 2018). Fleck and Kraemer (2014) proposed that any training could refer to the general definition of FT: the training aiming to increase performance for some functional task, such as activities of daily living or tests related to athletic performance. Considering that muscular strength, power, flexibility, and endurance are often considered functional aspects of the neuromuscular system, this general definition (Fleck and Kraemer, 2014) appears to be most rational.

The term FT originated in sports medicine and, more specifically, in rehabilitation clinics (Stenger, 2018). Early definitions focused on rehabilitation to enhance or develop the skills associated with activities of daily living and, frequently, when working with older adults (Stenger, 2018). In this context, the desired outcome is to restore (or rehabilitate) neuromuscular function. Guidelines and arguments for implementing FT for back pain prevention are essentially the same for back pain rehabilitation (Wirth et al., 2017). This is because the “functional” status of rehabilitation exercises is related to the activities and functions of the body and contextual factors such as environmental and personal factors (World Health Organization, 2013). Although strength and conditioning professionals are always working to improve a specific neuromuscular function, the term “FT” becomes redundant and confused.

All physical training programs have the purpose of improving or maintaining biological functions. The dissemination of these misconceptions can induce irreparable professional conduct. If FT is not a different training method from those already used in sports training, it is a classic case of needlessly reinventing the wheel. Therefore, we recommend that the term “functional training” no longer describe any physical training program.

## Author Contributions

BI conceived the idea, wrote the first draft, worked on all drafts, and formatted the manuscript for submission. MM, CS, AS, BS, MS, DO, and GM helped develop the main idea and draft the paper. All authors read and approved the final version of the manuscript.

## Funding

This research was funded by Federal University of Juiz de Fora, supporting the APC.

## Conflict of Interest

The authors declare that the research was conducted in the absence of any commercial or financial relationships that could be construed as a potential conflict of interest.

## Publisher's Note

All claims expressed in this article are solely those of the authors and do not necessarily represent those of their affiliated organizations, or those of the publisher, the editors and the reviewers. Any product that may be evaluated in this article, or claim that may be made by its manufacturer, is not guaranteed or endorsed by the publisher.
